# Polymorphisms in glucose homeostasis genes are associated with cardiovascular and renal parameters in patients with diabetic nephropathy

**DOI:** 10.1080/07853890.2022.2138531

**Published:** 2022-10-31

**Authors:** Sonia Mota-Zamorano, Luz M. González, Nicolás R. Robles, José M. Valdivielso, José C. Arévalo-Lorido, Juan López-Gómez, Guillermo Gervasini

**Affiliations:** a Department of Medical and Surgical Therapeutics, Medical School, Universidad de Extremadura, Badajoz, Spain; b RICORS2040 Renal Research Network, Madrid, Spain; c Service of Nephrology, Badajoz University Hospital, Badajoz, Spain; d Vascular and Renal Translational Research Group, UDETMA, IRBLleida, Lleida, Spain; e Service of Internal Medicine, Badajoz University Hospital, Badajoz, Spain; f Service of Clinical Analyses, Badajoz University Hospital, Badajoz, Spain; g Institute of Biomarkers of Molecular and Metabolic Pathologies, Universidad de Extremadura, Badajoz, Spain

**Keywords:** Chronic kidney disease, diabetic kidney disease, diabetic nephropathy, type 2 diabetes mellitus, glucose homeostasis

## Abstract

**Background:**

Diabetic nephropathy (DN) has become the major cause of end-stage kidney disease and is associated to an extremely high cardiovascular (CV) risk.

**Methods:**

We screened 318 DN patients for 23 SNPs in four glucose transporters (*SLC2A1*, *SLC2A2*, *SLC5A1* and *SLC5A2*) and in *KCNJ11* and *ABCC8*, which participate in insulin secretion. Regression models were utilised to identify associations with renal parameters, atherosclerosis measurements and CV events. In addition, 506 individuals with normal renal function were also genotyped as a control group.

**Results:**

In the patient’s cohort, common carotid intima media thickness values were higher in carriers of *ABCC8* rs3758953 and rs2188966 vs. non-carriers [0.78(0.25) vs. 0.72(0.22) mm, *p* < 0.05 and 0.79(0.26) vs. 0.72(0.22) mm, *p* < 0.05], respectively. Furthermore, *ABCC8* rs1799859 was linked to presence of plaque in these patients [1.89(1.03-3.46), *p* < 0.05]. Two variants, *SLC2A2* rs8192675 and *SLC5A2* rs9924771, were associated with better [OR = 0.49 (0.30-0.81), *p* < 0.01] and worse [OR = 1.92 (1.15-3.21), *p* < 0.05] CV event-free survival, respectively. With regard to renal variables, rs841848 and rs710218 in *SLC2A1*, as well as rs3813008 in *SLC5A2*, significantly altered estimated glomerular filtration rate values [carriers vs. non-carriers: 30.41(22.57) vs. 28.25(20.10), *p* < 0.05; 28.95(21.11) vs. 29.52(21.66), *p* < 0.05 and 32.03(18.06) vs. 28.14(23.06) ml/min/1.73 m^2^, *p* < 0.05]. In addition, *ABCC8* rs3758947 was associated with higher albumin-to-creatinine ratios [193.5(1139.91) vs. 160(652.90) mg/g, *p* < 0.05]. The epistasis analysis of SNP-pairs interactions showed that *ABCC8* rs3758947 interacted with several SNPs in *SLC2A2* to significantly affect CV events (*p* < 0.01). No SNPs were associated with DN risk.

**Conclusions:**

Polymorphisms in genes determining glucose homeostasis may play a relevant role in renal parameters and CV-related outcomes of DN patients.

## Introduction

Cardiovascular disease (CVD) is responsible for over sixty percent of the life-years lost from diabetes. Indeed, the risk of dying from CVD is two times higher for diabetics than for non-diabetic subjects [1]. Up to 40% of patients with diabetes develop diabetic nephropathy (DN), which has become the major cause of end-stage kidney disease (ESKD) worldwide, primarily because of the global obesity pandemic [2]. These DN patients are at such an elevated cardiovascular (CV) risk that is far more likely for them to die from CVD than from the deterioration of their renal function. The mechanisms underlying the solid link between DN and CVD are, however, poorly understood, and traditional CV risk factors, although commonplace in these patients, do not fully account for the observed elevated risk [3].

Diabetes has been consistently linked to macro and microvascular complications, mainly caused by hyperglycaemia, whose persistence results in increasing reactive oxygen species (ROS) generation, disruption of cell functioning and formation of abnormal proteins [4]. This hyperglycaemia is a key CV risk factor for patients with type 2 diabetes mellitus (T2DM). It has been reported that lowering of glucose levels reduce CV events (CVE) provided that (i) treatment is initiated early, (ii) hypoglycaemia is avoided and (iii) therapeutic regimes are individualised [5–7], although there are also some contradictory reports [8,9]. The advent of the SGLT2 (sodium/glucose cotransporter 2) inhibitors (SGLT2i), which increase urinary excretion of glucose as well as decreasing blood pressure (BP), has provided a major advance for the prevention and treatment of diabetes, CKD, and CV events, since the obtained decrease of albuminuria, prevents estimated glomerular filtration rate (eGFR) decline and reduces CV mortality [10,11].

A critical step in glucose homeostasis is its transport across the cells of the proximal tubule through two different classes of transporters, namely glucose transporters GLUT1 and 2, encoded by the *SLC2A1-2* genes, and sodium-dependent glucose transporters SGLT1 and 2, encoded by *SLC5A1-2* [12] (Supplementary Figure S1). Several SNPs in these loci, such as rs9934336, rs3813008, rs371505974 or rs200406921, have been related to impaired glucose homeostasis [13], the risk of DN [14,15] and CVD [16]. In addition, two proteins, namely Kir6.2 (potassium channel subunit) (*KCNJ11* gene) and SUR1 (sulfonylurea receptor-1) (*ABCC8* gene), form together an ATP-sensitive potassium channel that is key in glucose-induced insulin secretion by pancreatic beta cells (Supplementary Figure S1). Genetic variability in these two genes, most notably rs5219, has also been associated with glucose imbalance, T2DM and CVD [17–20].

Our aim was to target a set of genes that interact with each other to enable glucose reabsorption and insulin secretion by determining whether 23 common SNPs in their loci were associated with CV traits and events in DN patients. An additional objective was to identify variants related to DN risk.

## Patients and methods

### Participants

The present study was performed on patients and controls previously included in the NEFRONA repository. This was a collection of biological samples from Spanish CKD patients and healthy individuals who were recruited between 2010 and 2011 and were followed for 4 years to register atherosclerosis measurements and CV traits and events [21]. For this work, we focussed on a subgroup of CKD patients with DN. Our study was approved by the Scientific Committee of the NEFRONA group, which allowed us to screen samples from 318 patients with DN (stage 3 CKD or higher) and 506 controls with normal renal function for variants in genes related to glucose homeostasis.

Samples of the NEFRONA repository were analysed in this work if they corresponded to (i) patients over 18 years of age with T2DM (fasting glucose >126 mg/dL or non-fasting glucose >200 mg/dL) and deterioration of kidney function [eGFR <60 ml/min/1.73 m^2^ and >300 mg albumin (or >500 mg protein) in 24-hour urine]; or (ii) healthy subjects over 18 years of age with eGFR > 60 ml/min/1.73 m.^2^ The diagnosis of classical diabetic nephropathy was made using clinical criteria (proteinuria higher than 500 mg/day or microalbuminuria higher than 300 mg/day associated with documented diabetic retinopathy (confirmed by examining the back of the eye). A kidney biopsy was carried out to confirm the diagnosis in those cases where the patient did not present diabetic retinopathy and proteinuria was higher than 1 g/day, after patient consent was obtained. Other possible diagnoses were adequately ruled out using clinical protocols for glomerulopathy and immunological screening. NEFRONA exclusion criteria included previous history of any CV event, transplantation of any organ, carotid artery surgery, active infection, pregnancy, or life expectancy below one year.

A schematic representation of the study design is depicted in Supplementary Figure S2.

### Clinical variables

Diagnosis and staging of the CKD patients was carried out with the Kidney Disease Improving Global Outcomes (KDIGO) classification and the CONSORTIUM-CKD equation (www.kidneyriskfailure.org). Renal function was estimated with The Modification of Diet in Renal Disease (MDRD) equation.

High blood pressure was diagnosed according to 2013 ESH/ESC Guidelines for the management of arterial hypertension. Dyslipidemia diagnosis was made using the criteria of 2016 European Guidelines on Cardiovascular Disease prevention in clinical practice.

The presence of atheromatous plaques was assessed by arterial ultrasound as previously described [22] in ten different territories according to the Mannheim IMT Consensus guidelines [23] and the American Society of Echocardiography [24]. Three longitudinal measurements of intima media thickness (IMT) were carried out in the right and left common carotid arteries (CC). Atheromatous plaques were considered when IMT was >1.5 mm. CV risk was evaluated in a 4-year follow-up (54 months) during which the likelihood of experiencing CV events was recorded. Events considered were acute myocardial infarction, acute coronary syndrome, coronary catheterisation requiring angioplasty, coronary bypass, typical angina with positive stress tests, sudden death, cerebrovascular accident, and peripheral arterial disease. Aneurysm was also observed in the larger NEFRONA cohort of CKD patients, but no individuals with DN analysed in this work experienced this event.

All subjects gave written informed consent for their participation in the study, which was approved by the Ethics Committee of the Badajoz University Hospital (No 18002909), and it was carried out in accordance with the Declaration of Helsinki and its subsequent revisions.

### Genotyping

DNA for the genotyping analyses was obtained from whole-blood samples by QIAamp DNA Blood kits (Qiagen, Valencia, CA, USA) and stored at 4 °C in sterile plastic vials until analysis. Twenty-three SNPs in four genes coding for glucose transporters (*SLC2A1*, *SLC2A2*, *SLC5A1* and *SLC5A2*) and in two genes (*KCNJ11* and *ABCC8*) key for insulin secretion were studied. These SNPs were selected based on previous reports stating their influence on glucose homeostasis, T2DM, DN and/or associated CV traits [13,14,25–36]. Genetic analyses were carried out by allelic discrimination using a customised panel (TaqMan® OpenArray Genotyping) on a QuantStudio™ 12 K Flex Real-Time PCR System (Life Technologies, Carlsbad, CA, USA) at the Centro Nacional de Genotipado-Instituto de Salud Carlos III (CeGen-ISCIII; Madrid, Spain). Each run included a trio of Coriell Institute samples with known genotypes. Individuals with missing genotypes (ranging from 0.2% to 1.2% depending on the SNP) were ruled out in each analysis

### Statistical analyses

Categorical variables were compared with Chi-square test, whilst differences between continuous variables were assessed by T-Student’s/Mann–Whitney or ANOVA/Kruskal–Wallis, depending on the normality of the data and the numbers of groups compared. The effect of SNPs was evaluated by regression analyses adjusting by confounding variables that were selected based on univariate tests or by clinical criteria. Significant covariates used included demographics (age, sex, body-mass index) and clinical parameters, namely hyperlipidaemia, hypertension, and CKD stage. A dominant model of inheritance was chosen for the genetic analyses, as the resulting comparison groups were the most balanced in terms of size, as conducted in previous studies of our group with CKD patients [37,38]. The CV impact of the SNPs was analysed by Kaplan–Meier curves, comparing the different genotypes with the log-rank test. SNPs with p-values lower than 0.1 were included in Cox regression models to establish their effect after controlling for traditional CV risk factors. Patients were followed up until the earliest of CV event, death or end of study.

Gene-gene interaction (epistasis) analyses were performed by using log-likelihood ratio tests adjusted by relevant covariates in a dominant model. In the resulting plots, the diagonal line contains the P values from likelihood ratio test for the crude effect of each SNP, which are sorted by their genomic position. The upper triangle in the matrix contains the P values for the interaction (epistasis) log-likelihood ratio test. The lower triangle contains the P values from likelihood ratio test comparing the two-SNP additive likelihood to the best of the single-SNP models. The network visualisation of the gene-gene interactions was generated by Cytoscape software package v. 3.9.1.

Statistical analyses were performed with the *SNPassoc* (2.0-11) in the R environment v. 4.1.3 and with IBM SPSS software (SPSS Inc., Chicago, IL, USA, v. 22.0).

## Results

Main clinical and demographic characteristics of the population are described in Table 1. Median age (and interquartile range) was 57 (17) years for the control group and 63 (18) years for DN patients. The percentage of males was predominant in both groups (54.2 and 65.4%, respectively). Patients had significantly higher values of blood pressure and CCIMT and a higher incidence of hypertension, hyperlipidaemia, atherosclerotic plaques, and CV events experienced in the 4-year follow-up [median follow-up = 46.46 (4.11) months].

**Table 1.** Demographic and clinical characteristics of the population of study.

	Controls	DN
N	506	318
Age (yrs)	57 (17)	63 (18)***
Men	274 (54.2)	208 (65.4)**
BMI (kg)	27.8 (5.33)	29.12 (7.95)**
Albumin/Creatinine (mg/g)	7.2 (45.12)	186.41 (841.56)***
eGFR (ml/min/1.73m2)	89.19 (21.8)	29.43 (21.2)***
Stage of CKD		
3		120 (37.7)
4-5		125 (39.3)
Dialysis		73 (23.0)
Smoking		
Non-smoker	195 (38.5)	123 (38.7)
Current-smoker	106 (20.9)	66 (20.8)
′Former-smoker	205 (40.5)	129 (40.6)
Hypertension		
No	325 (64.2)	4 (1.3)
Yes	181 (35.8)	314 (98.7)***
Hyperlipidaemia		
No	327 (64.6)	58 (18.2)
Yes	179 (35.4)	260 (81.8)***
Pulse pressure (mmHg)	51 (16)	70 (27)***
Systolic blood pressure (mmHg)	132 (24)	150 (33)***
Dyastolic blood pressure (mmHg)	80 (14)	79.5 (16)
Triglycerides (mg/dL)	100 (68)	130 (89)***
Total cholesterol (mg/dL)	203.2 ± 34.74	170.38 ± 38.26*
LDL-Cholesterol (mg/dL)	126.68 ± 32.03	94.86 ± 34.17
HDL-Cholesterol (mg/dL)	51.1 (20)	44 (18)***
Calcium (mg/dL)	9.4 (0.55)	9.3 (0.74)***
Phosphorus (mg/dL)	3.49 (0.79)	4 (1.33)***
Calcium-phosphorus product	29.07 (47.44)	37.2 (11.15)***
Glycated haemoglobin (%)	5.6 (0.9)	7.1 (1.8)***
Fasting sugar (mg/dL)	97 (19)	139.5 (80)***
TyG	8.48 (0.80)	9.07 (0.96)***
Insulin	6 (1.2)	244 (76.7)***
ACEi	43 (8.5)	101 (31.8)***
ARBs	100 (19.8)	200 (62.9)***
Oral hypoglycaemic agents	45 (8.9)	102 (32.1)***
CCIMT (mm)	0.71 (0.21)	0.76 (0.23)***
CV Event		
No	497 (98.2)	253 (79.6)
Yes	9 (1.8)	65 (20.4)***
Presence of plaque		
No	229 (45.3)	57 (17.9)
Yes	277 (54.7)	261 (82.1)***

Data are shown as median (interquartile range), mean ± standard deviation or count and percentages in parenthesis.

BMI: body mass index; CCIMT: common carotid intimate media thickness; DN: diabetic nephropathy; eGFR: estimated glomerular filtration rate; TyG: Triglycerides and glucose index. ACEi: angiotensin converting enzyme inhibitors; ARBs: angiotensin II receptor blockers. *p < 0.05, **p < 0.01, ***p < 0.001.

Two out of the 23 SNPs, rs371505974 and rs200406921, were monomorphic in our population and hence were ruled out from further analyses. The remaining 21 SNPs did not show deviations from the Hardy-Weinberg equilibrium (*p* > 0.05 in all cases). The percentage of missing genotype data ranged from 0.2 to 1.2 depending on the specific SNP. Supplementary Table S1 summarises the main characteristics and predicted consequences of the SNPs studied. Statistical power calculations were carried out considering an arbitrary effect size of 2.0 for genetic variants and a type-I error of 0.05. With the reported incidence of the disease in the population [39] and the available sample size available, the statistical power of the study to identify genetic associations ranged from 0.821 to 0.944 depending on the minor allele frequency (Quanto v1.2.4, USC Los Angeles, USA)

### Risk analysis

Out of the 21 SNPs assayed, carriers of the rs3758947 A-allele in the *ABCC8* gene were at higher risk of DN than non-carriers [OR = 1.38 (1.02-1.89), *p* < 0.05]. However, after controlling for meaningful covariates, namely age, sex and BMI, the association lost statistical significance. None other risk associations were observed. Supplementary Table S2 shows the results of the risk analysis obtained for all the SNPs.

### Associations with renal function

**Figure 1.**
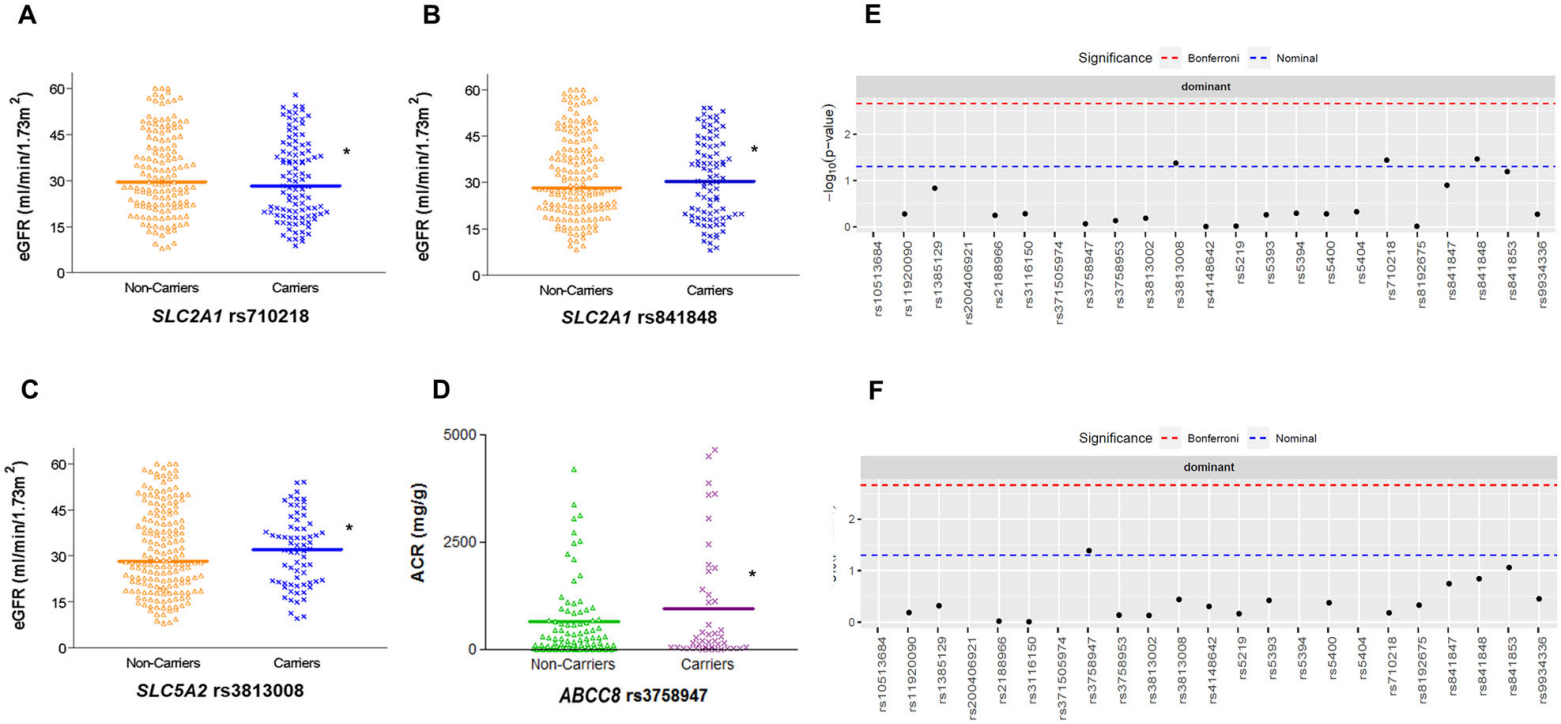
Estimated glomerular filtration rate (eGFR) values according to the presence of rs710218 (A), rs841848 (B) and rs3813008 (C) variants, and albumin-to-creatinine ratios (ACR) distributed by rs3758947 genotypes (D) in patients with diabetic nephropathy. Manhattan plots show the p-value for the association of all the SNPs studied regarding eGFR (E) and ACR (F). *p < 0.05.

We then analysed the cohort of 318 DN patients to identify associations with renal parameters. Linear regression analysis controlling for sex, hyperlipidaemia, CCIMT and CKD stage, showed that two SNPs in *SLC2A1* (coding for the GLUT1 transporter), rs841848 and rs710218, and the rs3813008 variant in *SLC5A2* (coding for SGLT2) were associated with eGFR values. Median (and interquartile range) values of carriers vs. non-carriers for these 3 SNPs were 30.41 (22.57) vs. 28.25 (20.10), *p* < 0.05; 28.95 (21.11) vs. 29.52 (21.66), *p* < 0.05 and 32.03 (18.06) vs. 28.14 (23.06) ml/min/1.73 m2, *p* < 0.05, respectively (Figure 1(A–C)). With regard to proteinuria, measured as albumin-to-creatinine ratios, *ABCC8* rs3758947 was significantly associated with higher median values in the DN patients [193.5 (1139.91) vs. 160 (652.90) mg/g creatinine, *p* < 0.05; Figure 1(D)]. Supplementary Table S3 shows the detailed statistical model obtained for these associations.

### Associations with atherosclerosis parameters and cardiovascular events

**Figure 2.**
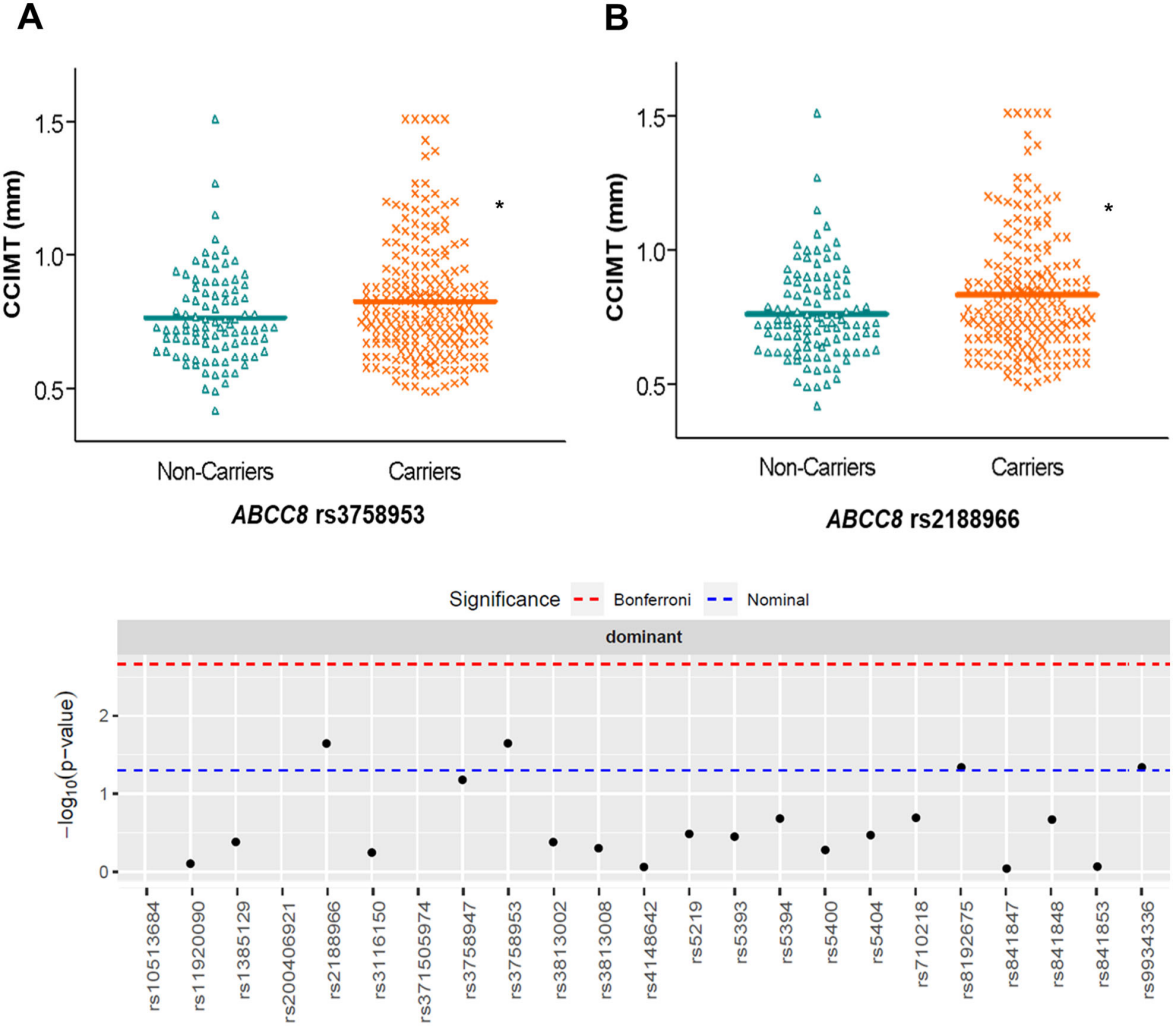
Distribution of common carotid intima media thickness (CCIMT) values according to different genotypes of *ABCC8* in patients with diabetic nephropathy. The Manhattan plot shows the p-value for the association of all the SNPs studied. *p < 0.05.

We examined genetic associations with measurements of atherosclerosis in the DN patients. After adjusting linear regression models for confounding factors (age, sex, hyperlipidaemia, hypertension, BMI and CKD stage), we observed that CCIMT median values were higher in carriers of two SNPs in *ABCC8*, rs3758953 and rs2188966, compared with wild-type subjects [0.78 (0.25) vs. 0.72 (0.22) mm, *p* < 0.05 and 0.79 (0.26) vs. 0.72 (0.22) mm, *p* < 0.05, respectively] (Figure 2 and Supplementary Table S4). We also found that *ABCC8* rs1799859 was associated with higher [OR = 1.89 (1.03-3.46), *p* < 0.05] risk of atherosclerotic plaque after controlling for confounding variables, whilst *KCNJ11* rs5219 showed a trend towards lower risk [OR = 0.54 (0.29-1.03), *p* < 0.05].

Regarding CV events, DN increased their incidence after adjusting for traditional risk factors. A total of 65 patients (20.4%) experienced these events in the four-year follow-up compared with only 9 subjects (1.8%) in the control group [OR = 5.39 (1.61-18.00), *p* < 0.05]. The proportion of patients affected within the DN cohort is inferior to other series reported, which can be explained by the fact that none of our patients had experienced CV events prior to their enrolment. Demographic and clinical features of DN patients who experienced or not CV events are shown in Table 2.

**Table 2.** Variables of interest in patients of diabetic nephropathy experiencing or not cardiovascular events.

	No CVE	CVE	*p* Value
N	253	65	
Age (yrs)	63 (19)	64 (15)	0.293
Sex			
Women	93 (36.8)	17 (26.2)	0.071
Men	160 (63.2)	48 (73.8)	
BMI (kg)	28.67 (8.54)	29.28 (5.48)	0.908
ACR (mg/g)	170.2 (818)	259.11 (2179)	0.526
eGFR (ml/min)	29.52 (21.25)	27.78 (20.17)	0.514
Stage of CKD			
3	98 (38.7)	22 (33.8)	0.244
4-5	102 (40.3)	23 (35.4)	
Dialysis	53 (20.9)	20 (30.8)	
Smoking			
Non-smoker	100 (39.5)	23 (35.4)	0.718
Current-smoker	53 (20.9)	13 (20)	
Former-smoker	100 (39.5)	29 (44.6)	
Hypertension			
No	2 (0.8)	2 (3.1)	0.187
Yes	251 (99.2)	63 (96.9)	
Hyperlipidaemia			
No	46 (18.2)	12 (18.5)	0.542
Yes	207 (81.8)	53 (81.5)	
Pulse pressure (mmHg)	71.1 ± 20	73.23 ± 20.52	0.723
Systolic lood pressure (mmHg)	150.04 ± 23.54	153.54 ± 25.63	0.635
Dyastolic blood pressure (mmHg)	78.94 ± 11.50	80.31 ± 12.87	0.471
Total cholesterol (mg/dL)	168 (49)	164 (60)	0.803
Triglycerides and glucose index	9.11 ± 0.78	9.13 ± 0.71	0.458
CCIMT	0.76 (0.21)	0.83 (0.30)	*p* < 0.05
Presence of plaque			
No	53 (20.9)	4 (6.2)	*p* < 0.01
Yes	200 (79.1)	61 (93.8)	
Glucose (mg/dL)	139 (81)	149 (77)	0.706
Calcium (mg/dL)	9.3 (0.71)	9.1 (0.8)	0.453
Sodium (mEq/L)	140 (3)	140 (4)	0.662
Potassium (mEq/L)	4.92 ± 0.6	5.11 ± 0.63	0.496

ACR: albumin-to-creatinin ratio; CCIMT: common carotid intimate media thickness.

**Figure 3.**
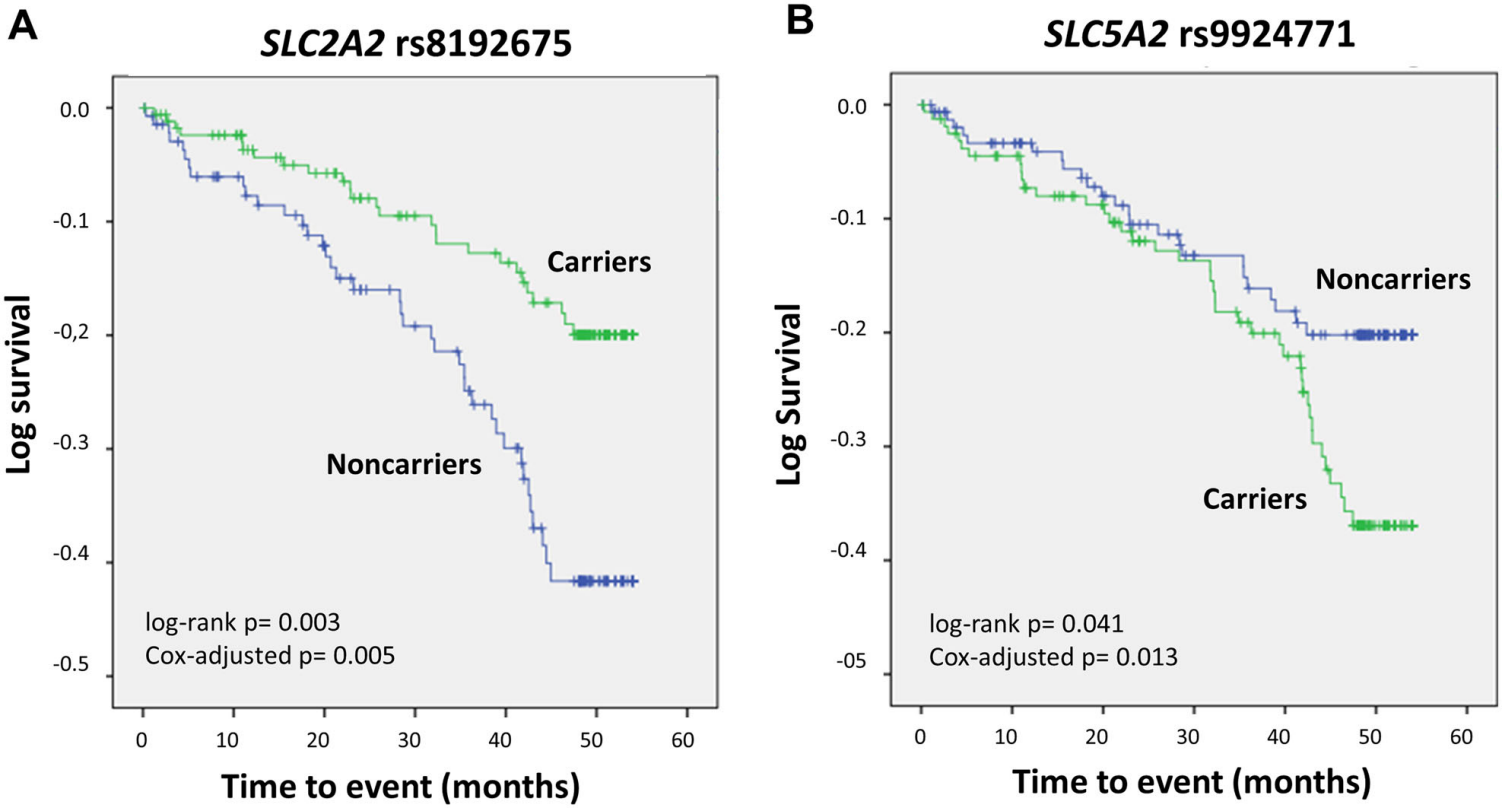
Kaplan–Meier curves depicting cardiovascular event-free survival in patients with diabetic nephropathy carrying the *SLC2A2* rs8192675 (A) or *SLC5A2* rs9924771 (B) polymorphisms.

Genetic analyses to determine associations with CV events in the control group could not be tested, as only 9 subjects experienced CV events. Survival analysis in the DN cohort revealed two SNPs with a significant impact. *SLC2A2* rs8192675 was associated with an increased estimated CV event-free median (interquartile range) survival [49.19 (0.96) months compared with 44.69 (1.38) for the wild-type genotype; log-rank *p* < 0.01]. In contrast, patients harbouring the *SLC5A2* rs9924771 SNP presented lower survival than non-carriers did [46.27 (1.18) vs. 48.40 (1.11) months, log-rank *p* < 0.05]. Figure 3 shows Kaplan-Meier curves depicting event-free survival for each of the mentioned genotypes. The statistical significance of these associations was maintained after controlling for traditional CV risk factors in Cox regression models, which resulted in OR values of 0.49 (0.30-0.81), *p* < 0.01 and 1.92 (1.15-3.21), *p* < 0.05 for rs8192675 and rs9924771, respectively.

Table 3 shows the estimated impact on event-free survival of a regression model including both SNPs simultaneously.

**Table 3.** Cox regression analysis showing the effect of rs8192675 and rs9924771 on cardiovascular event-free survival in diabetic nephropathy patients.

	B	SE	Wald	OR	CI	*p* Value
rs8192675	−0.69	0.26	7.27	0.50	0.30-0.83	0.007
rs9924771	0.631	0.26	5.76	1.90	1.14-3.19	0.015
Age	0.012	0.01	1.09	1.01	0.99-1.3	0.297
Sex	0.461	0.30	2.43	1.59	0.89-2.83	0.119
BMI	−0.033	0.03	1.85	0.97	0.92-1.02	0.173
Hyperlipidaemia	0.156	0.33	0.23	1.17	0.62-2.22	0.634
CKD stage	0.533	0.18	8.93	1.71	1.20-2.42	0.003
Hypertension	−0.952	0.77	1.52	0.39	0.09-1.76	0.218

BMI: body mass index; CKD: chronic kidney disease.

### Multiple-SNP analysis

**Figure 4.**
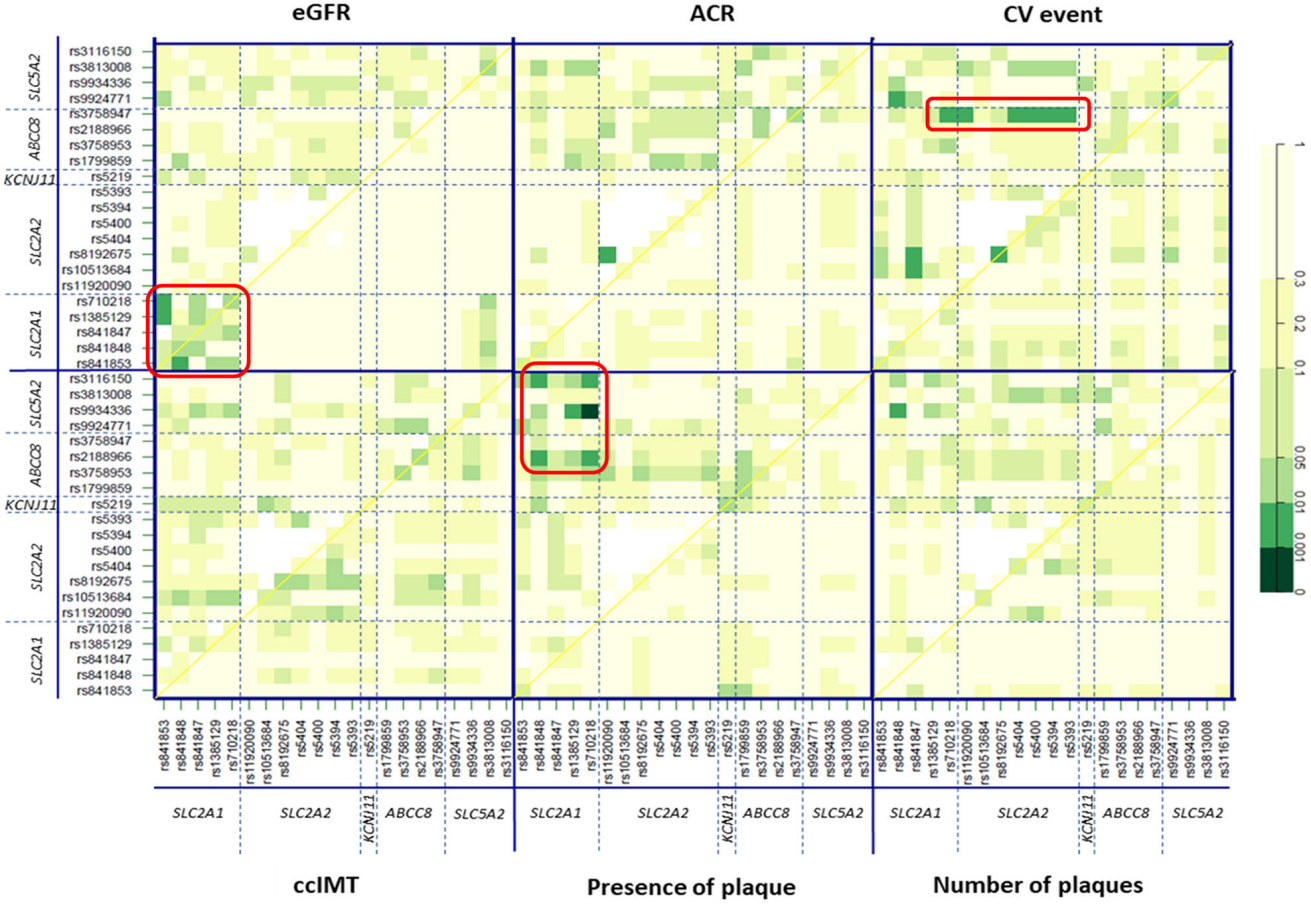
Effect of interactions between SNPs in glucose homeostasis genes on several clinical phenotypes in CKD patients. Significant hits discussed in the text are highlighted in red. The diagonal line contains the P values from likelihood ratio test for the crude effect of each SNP, which are sorted by their genomic position. The upper triangle in the matrix contains the P values for the interaction (epistasis) log-likelihood ratio test. Finally, the lower triangle contains the P values from likelihood ratio test comparing the two-SNP additive likelihood to the best of the single-SNP models. eGFR: estimated glomerular filtration rate (ml/min/1.73 m^2^); ACR: albumin-to-creatinine ratio (mg/g); CV: cardiovascular; CCIMT: common carotid intima media thickness.

We finally conducted an epistasis study to evaluate interactions between SNP pairs in the genes involved in glucose homeostasis. In relation to parameters of renal function, a quadrant corresponding to significant associations between pairs located within the *SLC2A1* gene locus and eGFR estimation was clearly noticeable (p-values ranging between <0.05 and <0.01; Figure 4). In addition, the rs8192675-rs11920090 pair in *SLC2A2* showed a far higher influence on albumin-to-creatinine (ACR) ratios (*p* < 0.01) than that of these SNPs by separate (Figure 4). Regarding CV events, *ABCC8* rs3758947 was observed to interact significantly (*p*-values <0.01) with several consecutive polymorphisms in *SLC2A2* (Supplementary Table S5 shows specific p-values for all the SNP-SNP interactions considering CV risk). Finally, the presence of plaque was mostly associated with SNP pairs in the top left quadrants, thus indicating interactions between *SLC5A2*-*SLC2A1* and *SLC5A2-ABCC8*. The highest significance was observed for the *SLC5A2* rs9934336-*SLC2A1* rs710218 pair in relation to the presence of plaque (*p* < 0.001; Figure 4). Supplementary Figure S3 shows a network visualisation of the discussed interactions.

## Discussion

Patients with DN not only show a disproportionately higher risk of CVD than the general population, but this risk is also greatly elevated in comparison with diabetics who have normal renal function [40]. Given the limitations of current DN biomarkers such as albumin urinary excretion, the identification of new markers of the disease, particularly for its CV implications, has been pointed out as essential for risk stratification, individualisation of treatment and identification of novel therapeutic targets [41–43]. In the present study, we have focussed on the role that variants in loci related to glucose homeostasis may play in this regard.

Our findings show that several SNPs in the *KCNJ11* and *ABCC8* genes, both coding for subunits of the ATP-sensitive potassium channel (KATP) that participates in insulin secretion [44], were associated with subclinical atherosclerosis (as shown by CCIMT values) and with the presence of atherosclerotic plaque in the CKD patients. These two genes, which are located on the same chromosome locus 11p15.1, are only 4.5 kb apart and indeed genetic variants in both loci have been reported to cause similar phenotypes. Several SNPs in *KCNJ11* were initially reported to be linked to T2DM susceptibility [45] and subsequently to vascular-related complications of T2DM such as hypertension [46], coronary artery disease (CAD) [47] or diabetic retinopathy [48]. We observed that rs5219, a nonsynonymous (Glu23Lys) SNP, showed a trend towards lower presence of plaque. In line with this finding, the variant allele in this locus has also been related to decreased risk of CVD, both in the general population [49] and in T2DM patients [50]. However, no previous studies have been carried out in DN patients, nor have there been studies aimed to determine the impact of rs5219 on atherosclerosis measurements.

According to our findings, the most significant gene for atherosclerosis was *ABCC8*, with two relevant variants in the 5′-UTR region (rs2188966 and rs3758953) and a synonymous AGG1273AGA SNP (rs1799859). This locus is also known to harbour numerous mutations reportedly affecting insulin secretion through impairment of the KATP channel, which may eventually lead to hyperglycaemia and a wide spectrum of T2DM phenotypes [51]. Specifically, the SNPs studied in the present work had been previously associated with T2DM susceptibility [25,52] and response to sulfonylurea treatment [53]. Only a very recent study by Gurzeler et al. [54] has analysed the effect of one other *ABCC8* SNP (Glu1506Lys) on atherosclerosis, although this was published after the completion of our work and hence we could not include the SNP in our analyses. These authors found that the 1506Lys variant impaired glucose tolerance and increased arterial inflammation, thus promoting a vulnerable atherosclerotic plaque phenotype in rodents. It is tempting to speculate that other SNPs in the same locus, such as those described herein, may also promote plaque formation through similar mechanisms.

Two SNPs, rs8192675 and rs9924771, displayed a significant impact on CV event-free survival after other traditional risk factors were considered. The first is an intronic variant (transcript NM_000340.2:c.612 + 54T > C) in *SLC2A2*, which encodes the facilitated glucose transporter GLUT2, and that has been consistently linked to better response to metformin monotherapy in T2DM patients [27,55]. Available gene expression data point to a reduction in GLUT2 expression caused by this rs8192675 [55], but its full functional consequences are still unclear and hence we cannot establish a direct biochemical connection with the observed improved survival. One study has reported that the minor allele was associated with lower levels of high-density lipoproteins in hypertensive subjects, and the authors suggested this could increase metabolic risk of CVD, although this was not confirmed [56]. In contrast, in our study, the minor allele was associated with a 5-month improved CV event-free survival. It should be noted that we did not observe the reported impact of rs8192675 on HDL in our cohort (data not shown). Hence, additional *in vitro* studies are necessary to unveil other possible mechanisms, e.g. effect on glycaemia or insulin secretion, that may explain why this SNP impacts CV outcomes significantly in CKD patients [57]. The second polymorphism, rs9924771, is located in an intronic region of *SLC5A2*, a gene that codes for the SGLT2 transporter. This is a tag-SNP, meaning that it represents variability of a whole region in the gene locus [28]. To our knowledge, there have been no studies on the effect of this variant on CV outcomes in CKD. On the other hand, and consistent with our results, variability in the *SLC5A2* gene has been recently regarded as an important locus for CVD in a large study with over 400,000 participants [16]. The underlying mechanism remains elusive though, as neither these authors, nor the present work, could find significant associations with atherosclerotic measurements. Furthermore, other reports have not found an impact of rs9924771 on insulin release, plasma glucose or plasma glucagon concentrations [28,58], although it should be noted that we did observe a trend towards higher HbA1c levels in rs9924771 carriers (*p* = 0.05, data not shown). In any case, even though rs8192675 and rs9924771 correlate with CV outcomes in DN patients, causality is still to be confirmed until an explanatory mechanism has been identified.

The analysis of the effect of SNP pairs interactions on CV traits revealed an intriguing association between *ABCC8* rs3758947 and several SNPs mainly located in *SLC2A2*, which modified the risk of CV events. To our knowledge, there are no studies on the combined effect of SNPs in these genes, but our data show that this interaction could have clinical relevance. The interacting genes code for GLUT2 and SUR1, which work together in pancreatic cells for insulin secretion; therefore, it is tempting to speculate that the combined presence of functional variants could impair insulin release and affect CV phenotypes. The most significant gene-gene interaction regarding CV traits was *SLC5A2* rs9934336- *SLC2A1* rs710218, which was associated with an increased risk of atherosclerotic plaque formation. However, it should be noted that these genes had not shown a relevant effect on atherosclerosis in the single-SNP approach and hence this association, although it showed a p-value <0.001, should be interpreted with caution.

The study of renal function in the DN cohort revealed that SNPs in *SLC2A1* (GLUT1) and *SLC5A2* (SGLT2) were associated with eGFR values after adjusting by CKD stage and other variables. However, the fact that these variants did not affect DN risk raises questions about their clinical relevance. These differences were more noticeable for the *SLC5A2* rs3813008 variant, an intronic tag-SNP that has been studied in relation to T2DM susceptibility and glycaemic parameters [14,58], but that to date was untested in the CKD setting. The *SLC5A2*-encoded SGLT2 transporter has been widely studied because of the advent of SGLT2i, which have evolved from glucose-lowering drugs to renoprotective and cardioprotective agents potentially useful in patients with low eGFR [59]. However, and somewhat surprisingly, there are no previous data on the impact of genetic variants in the gene locus (which could somehow mimic the effect of SGLT2i) on renal function. Our results, together with the aforementioned association with CV outcomes in our DN patients suggest that the determination of *SLC5A2* genetic variability could be useful in renal patients and a factor to bear in mind when prescribing SGLT2i. As for proteinuria, a hallmark of DN, we observed that carriers of the *ABCC8* rs3758947 variant allele had, on average, 268 mg albumin/g creatinine in urine more than wild type patients. Polymorphisms in this gene have been related to T2DM risk because of hyperglycaemia derived from impaired insulin secretion [25]. Although this could be a plausible hypothesis for the association with higher proteinuria, we could not confirm that levels of glucose correlated with the SNP in our cohort (data not shown) and therefore the processes explaining this association are still unclear.

Finally, we analysed a cohort of healthy subjects to identify variants that could be associated with the risk of DN. However, we could not identify relevant SNPs in this regard after adjusting for other risk factors. This result likely stems from a limitation of the study, which is that only 55 of the 506 controls with normal renal function had T2DM. A larger group of fully characterised T2DM patients without CKD would have been desirable to perform risk analyses. Another limitation of the study is that clinical associations lost statistical significance after correction for multiple testing. Furthermore, observed differences of eGFR and proteinuria across genotypes, although statistically significant, might not be as large as to have clinical relevance. Studies in groups with a higher proportion of patients with advanced CKD might provide with a wider range of eGFR and proteinuria values and hence differences according to genotype would presumably be more noticeable.

In summary, our findings indicate that polymorphisms in genes coding for proteins and transporters key for glucose homeostasis may affect CV-related outcomes in patients with DN. Specifically, polymorphisms in the two subunits that form the KATP channel regulating insulin secretion were associated with atherosclerosis measurements in these subjects, which supports the proposed link between T2DM and atherosclerosis [60]. In addition, variability in GLUT2, also involved in insulin secretion, and in the glucose transporter SGLT2, resulted in altered CV event-free survival, which is particularly relevant in a pathology such as DN, which confers an exceptionally high CV risk. On the other hand, the reported differences observed for renal function in our cohort warrant additional, larger studies that can compare patients showing a wide range of eGFR values.

## Supplementary Material

Supplemental Material

Supplemental Material

Supplemental Material

Supplemental Material

## Data Availability

The datasets underlying this article can be found at Figshare with DOI 10.6084/m9.figshare.20936788
