# Supplementary figures and images for: Polymorphisms in glucose homeostasis genes are associated with cardiovascular and renal parameters in patients with diabetic nephropathy
Source: Ann Med. 2022 Oct 31;54(1):3039–51. doi: 10.1080/07853890.2022.2138531 (PMC9635471; doi:10.1080/07853890.2022.2138531)

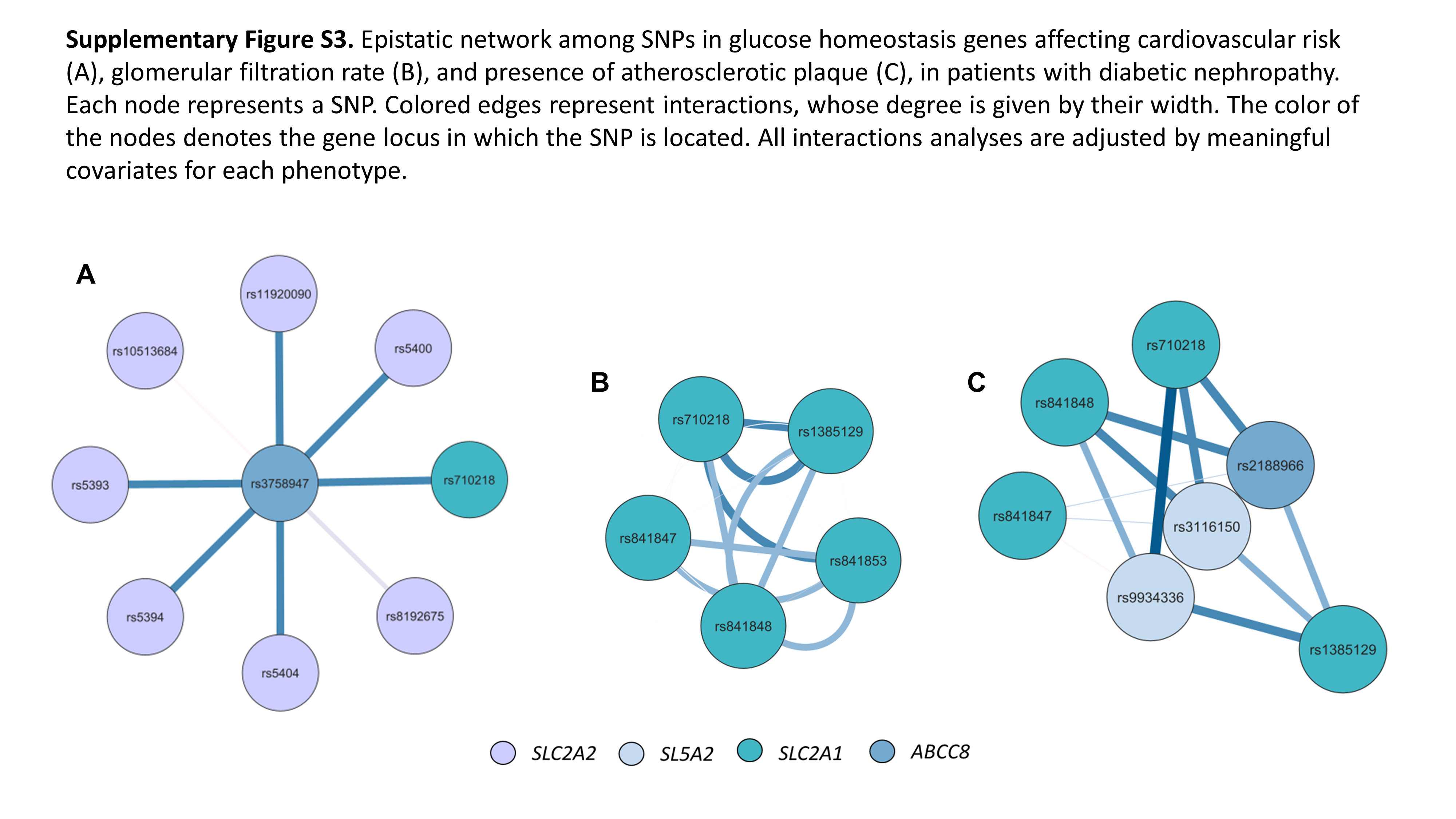

Supplement: Supplemental Material [file IANN_A_2138531_SM6417.tif]

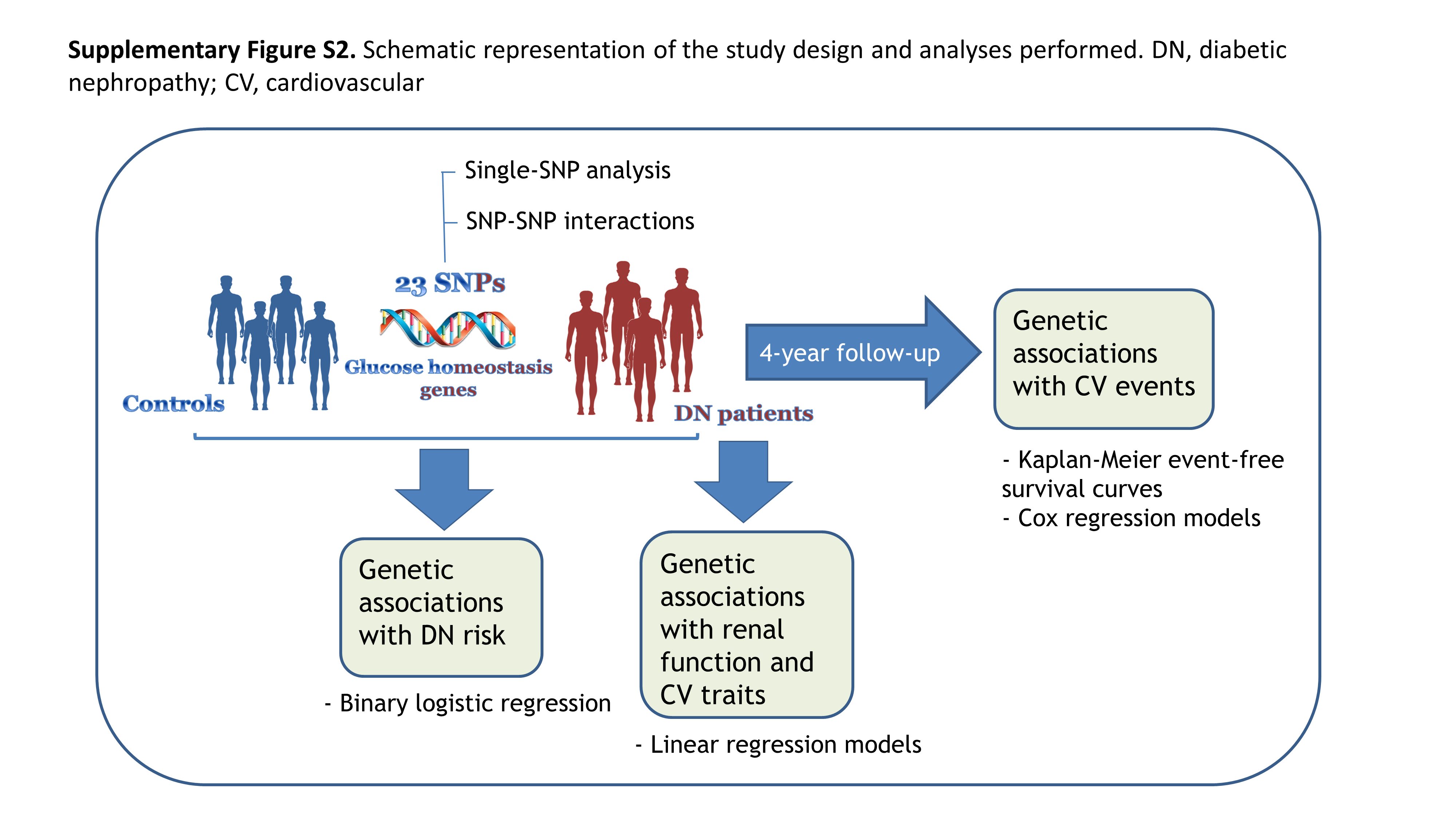

Supplement: Supplemental Material [file IANN_A_2138531_SM6408.tif]

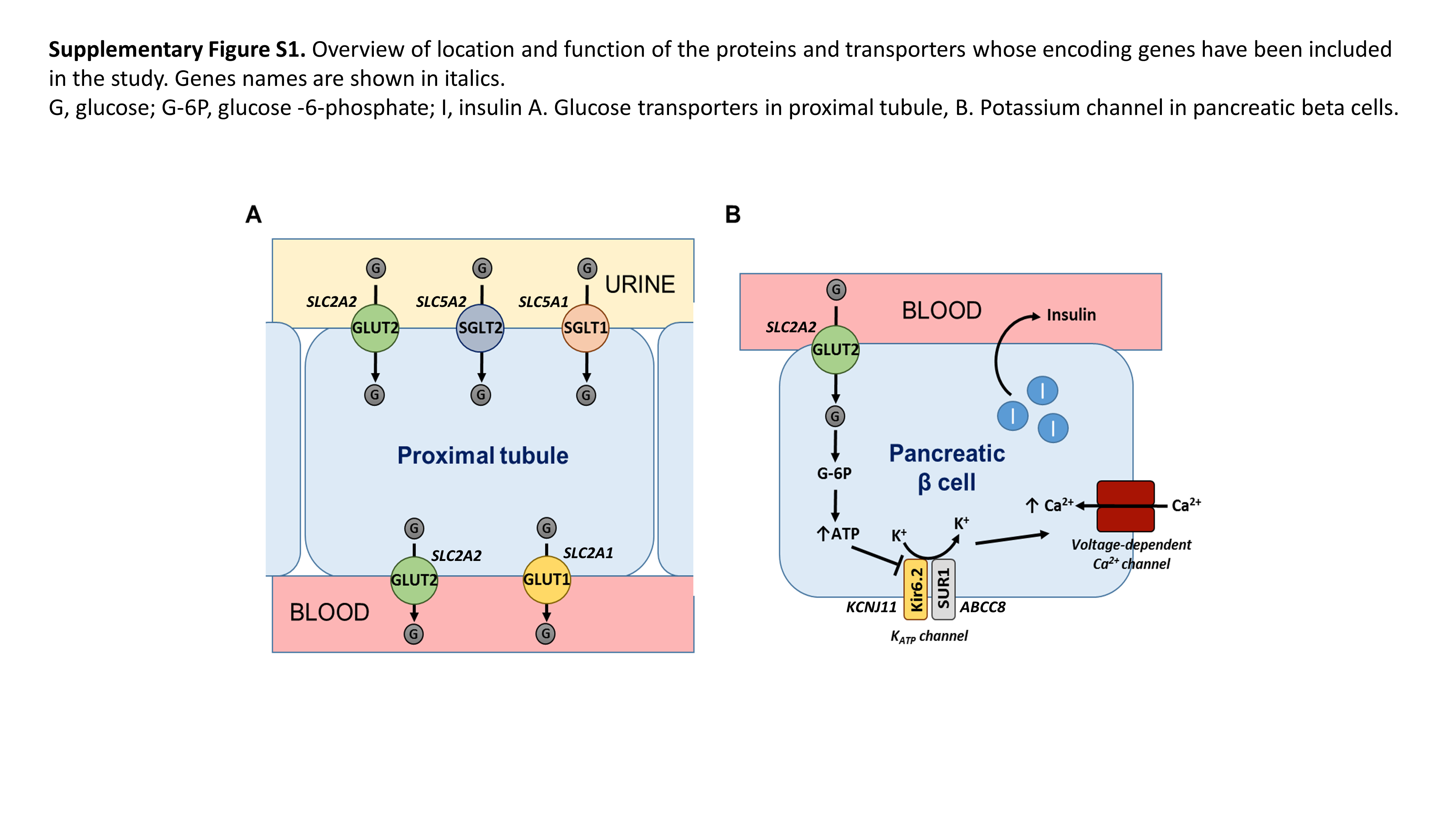

Supplement: Supplemental Material [file IANN_A_2138531_SM6401.tif]
