# Supplementary material for: Polymorphisms in glucose homeostasis genes are associated with cardiovascular and renal parameters in patients with diabetic nephropathy
Source: Ann Med. 2022 Oct 31;54(1):3039–51. doi: 10.1080/07853890.2022.2138531 (PMC9635471; doi:10.1080/07853890.2022.2138531)
Supplement: Supplemental Material [file IANN_A_2138531_SM6389.docx]

**Supplementary Table S1.** List of genetic polymorphisms included in the present study.

| **rs code** | **Gene (protein)** | **Position** | **Ref allele** | **Alternate allele** | **Functionality** | **Predicted consequence** | **HWE** |
| --- | --- | --- | --- | --- | --- | --- | --- |
| rs841853 | *SLC2A1* (GLUT1) | 1:42935767 | C | A | Intron variant | Benign | 0.940 |
| rs841848 | *SLC2A1* (GLUT1) | 1:42936874 | G | A | Intron variant | Benign | 0.490 |
| rs841847 | *SLC2A1* (GLUT1) | 1:42937037 | C | T | Intron variant | *Deleterious potential | 0.615 |
| rs1385129 | *SLC2A1* (GLUT1) | 1:42943295 | G | A | Synonymous variant | Benign | 1.000 |
| rs710218 | *SLC2A1* (GLUT1) | 1:42961547 | T | A | Intron variant | Benign | 0.759 |
| rs11920090 | *SLC2A2* (GLUT2) | 3:170999732 | T | A | Intron variant | Benign | 0.679 |
| rs10513684 | *SLC2A2* (GLUT2) | 3:171006421 | C | T | Intron variant | Benign | 0.162 |
| rs8192675 | *SLC2A2* (GLUT2) | 3:171007094 | T | C | Intron variant | Benign | 0.752 |
| rs5404 | *SLC2A2* (GLUT2) | 3:171007166 | C | T | Synonymous variant | Benign | 0.442 |
| rs5400 | *SLC2A2* (GLUT2) | 3:171014511 | G | A | Missense variant | Benign | 0.495 |
| rs5394 | *SLC2A2* (GLUT2) | 3:171027104 | G | A | Upstream transcript variant | Benign | 0.281 |
| rs5393 | *SLC2A2* (GLUT2) | 3:171027131 | T | G | Upstream transcript variant | Benign | 0.891 |
| rs5219 | *KCNJ11 (*Kir6*.2)* | 11:17388025 | C | T | Missense variant | Benign | 0.160 |
| rs1799859 | *ABCC8 (*SUR1*)* | 11:17397732 | G | A | Intron variant | 3'UTR variant | 0.109 |
| rs3758953 | *ABCC8 (*SUR1*)* | 11:17478000 | A | G | Upstream transcript variant | Benign | 0.325 |
| rs2188966 | *ABCC8 (*SUR1*)* | 11:17478338 | C | T | Upstream transcript variant | Benign | 0.659 |
| rs3758947 | *ABCC8 (*SUR1*)* | 11:17479663 | G | A | Intron variant | *Deleterious potential | 0.284 |
| rs9924771 | *SLC5A2 (*SGLT2*)* | 16:31484256 | C | T | Intron variant | *Deleterious potential | 0.861 |
| rs9934336 | *SLC5A2 (*SGLT2*)* | 16:31484552 | G | A | Intron variant | Benign | 0.401 |
| rs3813008 | *SLC5A2 (*SGLT2*)* | 16:31486560 | G | A | Intron variant | Benign | 0.763 |
| rs3116150 | *SLC5A2 (*SGLT2*)* | 16:31486700 | G | A | Intron variant | Benign | 0.088 |
| rs371505974 | *SLC5A2 (*SGLT2*)* | 22:32084966 | G | G | Missense variant | Deleterious | - |
| rs200406921 | *SLC5A2 (*SGLT2*)* | 22:32099305 | C | C | Missense variant | Deleterious | - |

Combined Annotation Dependent Depletion (CAAD) scores for these variants were between 10 and 15. Deleteriousness threshold is between 10 and 20

# Supplementary Table S2. P-values for the risk analysis in three models of inheritance

| **Gene (protein)** | **rs code** | **codominant** | **dominant** | **recessive** |
| --- | --- | --- | --- | --- |
| *SLC2A1* (GLUT1) | rs841853 | 0.365 | 0.439 | 0.383 |
| *SLC2A1* (GLUT1) | rs841848 | 0.922 | 0.949 | 0.710 |
| *SLC2A1* (GLUT1) | rs841847 | 0.510 | 0.591 | 0.253 |
| *SLC2A1* (GLUT1) | rs1385129 | 0.560 | 0.397 | 0.390 |
| *SLC2A1* (GLUT1) | rs710218 | 0.240 | 0.144 | 0.233 |
| *SLC2A2* (GLUT2) | rs11920090 | 0.565 | 0.690 | 0.289 |
| *SLC2A2* (GLUT2) | rs10513684 | 0.970 | - | - |
| *SLC2A2* (GLUT2) | rs8192675 | 0.285 | 0.199 | 0.626 |
| *SLC2A2* (GLUT2) | rs5404 | 0.393 | 0.578 | 0.265 |
| *SLC2A2* (GLUT2) | rs5400 | 0.423 | 0.919 | 0.216 |
| *SLC2A2* (GLUT2) | rs5394 | 0.519 | 0.784 | 0.299 |
| *SLC2A2* (GLUT2) | rs5393 | 0.889 | 0.705 | 0.839 |
| *KCNJ11 (*Kir6.2*)* | rs5219 | 0.721 | 0.775 | 0.540 |
| *ABCC8 (*SUR1*)* | rs1799859 | 0.186 | 0.467 | 0.181 |
| *ABCC8 (*SUR1*)* | rs3758953 | 0.302 | 0.818 | 0.176 |
| *ABCC8 (*SUR1*)* | rs2188966 | 0.220 | 0.315 | 0.094 |
| *ABCC8 (*SUR1*)* | rs3758947 | 0.305 | 0.124 | 0.720 |
| *SLC5A2 (*SGLT2*)* | rs9924771 | 0.190 | 0.101 | 0.798 |
| *SLC5A2 (*SGLT2*)* | rs9934336 | 0.837 | 0.829 | 0.644 |
| *SLC5A2 (*SGLT2*)* | rs3813008 | 0.833 | 0.564 | 0.966 |
| *SLC5A2 (*SGLT2*)* | rs3116150 | 0.472 | 0.255 | 0.965 |

**Supplementary Table S3**. Statistical models obtained for the genetic associations with renal parameters depicted in Figure 1

| **eGFR** | | | | | | |
| --- | --- | --- | --- | --- | --- | --- |
| **Gene** | **Polymorphism** | **Genotype** | **n (mean)** | **mean difference** | **p-value** | **R-square** |
| *SLC2A1* | rs841848 | G/G | 156 (31.96) | -1.81 (-3.69 -0.06) | 0.042 | 73.7% |
|  |  | G/A-A/A | 81 (30.45) |  |  |  |
|  | rs710218 | T/T | 140 (32.38) | -1.78 (-3.59 -0.03) | 0.046 | 74.1% |
|  |  | T/A-A/A | 96 (30.04) |  |  |  |
| *SLC5A2* | rs3813008 | G/G | 172 (31.52) | -2.39 (-4.42 -0.36) | 0.038 | 73.7% |
|  |  | G/A-A/A | 64 (31.45) |  |  |  |
| **ACR** | | | | | | |
| **Gene** | **Polymorphism** | **Genotype** | **n (mean)** | **mean difference** | **p-value** | **R-square** |
| *ABCC8* | rs3758947 | G/G | 96 (641.09) | 485.9 (29.96 -941.80) | 0.038 | 7.1% |
|  |  | G/A-A/A | 43 (933.63) |  |  |  |

**Supplementary Table S4.** Statistical models obtained for the genetic association with CCMIT values depicted in Figure 2

| **ccIMT (mm)** | | | | | | |
| --- | --- | --- | --- | --- | --- | --- |
| **Gene** | **Polymorphism** | **Genotype** | **n (mean)** | **mean difference** | **p-value** | **R-square** |
| *ABCC8* | rs3758953 | A/A | 94 (0.76) | 0.049 (0.003 0.096) | 0.037 | 19.7% |
|  |  | A/G-G/G | 212 (0.83) |  |  |  |
|  | rs2188966 | C/C | 108 (0.76) | 0.048 (0.003 0.093) | 0.038 | 19.4% |
|  |  | C/T-T/T | 198 (0.83) |  |  |  |

**Supplementary Table S5.** P-values obtained for the association of SNP-SNP interactions with cardiovascular risk in diabetic nephropathy patients

|  | rs841853 | rs841848 | rs841847 | rs1385129 | rs710218 | rs11920090 | rs10513684 | rs8192675 | rs5404 | rs5400 | rs5394 | rs5393 | rs5219 | rs4148642 | rs3758953 | rs2188966 | rs3758947 | rs3813002 | rs9934336 | rs3813008 | rs3116150 |
| --- | --- | --- | --- | --- | --- | --- | --- | --- | --- | --- | --- | --- | --- | --- | --- | --- | --- | --- | --- | --- | --- |
| rs841853 | 0.206 | 0.995 | - | 0.823 | 0.323 | 0.563 | 0.009 | 0.049 | 0.194 | 0.397 | 0.225 | 0.369 | 0.526 | 0.672 | 0.571 | 0.906 | 0.451 | 0.445 | 0.389 | 0.156 | 0.828 |
| rs841848 | 0.348 | 0.158 | 0.845 | 0.318 | 0.990 | 0.566 | 0.205 | 0.410 | 0.897 | 0.791 | 0.817 | 0.569 | 0.312 | 0.230 | 0.163 | 0.451 | 0.133 | 0.002 | 0.027 | 0.976 | 0.578 |
| rs841847 | 0.971 | 0.466 | 0.332 | 0.419 | 0.647 | 0.300 | 0.001 | 0.004 | 0.089 | 0.167 | 0.113 | 0.174 | 0.114 | 0.435 | 0.939 | 0.571 | 0.224 | 0.013 | 0.820 | 0.019 | 0.419 |
| rs1385129 | 0.853 | 0.390 | 0.819 | 0.647 | - | 0.919 | 0.091 | 0.596 | 0.772 | 0.850 | 0.831 | 0.939 | 0.738 | 0.628 | 0.040 | 0.152 | 0.181 | 0.613 | 0.575 | 0.350 | 0.128 |
| rs710218 | 0.379 | 0.963 | 0.429 | 0.180 | 0.301 | 0.604 | 0.291 | 0.165 | 0.735 | 0.680 | 0.673 | 0.492 | 0.381 | 0.891 | 0.024 | 0.150 | 0.018 | 0.252 | 0.576 | 0.357 | 0.103 |
| rs11920090 | 0.240 | 0.151 | 0.340 | 0.637 | 0.297 | 0.204 | - | 0.379 | - | - | - | 0.308 | 0.679 | 0.305 | 0.841 | 0.734 | 0.017 | 0.818 | 0.531 | 0.053 | 0.844 |
| rs10513684 | 0.565 | 0.440 | 0.508 | 0.628 | 0.457 | 0.960 | 0.522 | - | - | - | - | - | 0.729 | 0.486 | 0.908 | 0.650 | 0.460 | 0.217 | 0.287 | 0.048 | 0.753 |
| rs8192675 | 0.262 | 0.123 | 0.349 | 0.619 | 0.318 | 0.683 | 0.798 | 0.007 | - | - | - | 0.383 | 0.141 | 0.411 | 0.438 | 0.470 | 0.183 | 0.887 | 0.959 | 0.887 | 0.124 |
| rs5404 | 0.395 | 0.264 | 0.351 | 0.607 | 0.291 | 0.543 | 0.912 | 0.525 | 0.326 | - | - | 0.107 | 0.747 | 0.503 | 0.724 | 0.596 | 0.013 | 0.968 | 0.928 | 0.064 | 0.893 |
| rs5400 | 0.218 | 0.119 | 0.325 | 0.586 | 0.266 | 0.820 | 0.876 | 0.814 | 0.125 | 0.167 | - | 0.101 | 0.739 | 0.417 | 0.527 | 0.446 | 0.013 | 0.968 | 0.916 | 0.064 | 0.966 |
| rs5394 | 0.458 | 0.305 | 0.403 | 0.615 | 0.338 | 0.431 | 0.848 | 0.453 | 0.379 | 0.078 | 0.374 | - | 0.878 | 0.426 | 0.659 | 0.536 | 0.016 | 0.886 | 0.864 | 0.055 | 0.968 |
| rs5393 | 0.264 | 0.153 | 0.299 | 0.549 | 0.243 | 0.942 | 0.982 | 0.715 | 0.640 | 0.667 | 0.178 | 0.224 | 0.979 | 0.402 | 0.599 | 0.503 | 0.020 | 0.739 | 0.852 | 0.025 | 0.941 |
| rs5219 | 0.211 | 0.154 | 0.325 | 0.684 | 0.306 | 0.252 | 0.623 | 0.126 | 0.378 | 0.196 | 0.447 | 0.288 | 0.129 | 0.674 | 0.615 | 0.622 | 0.734 | 0.544 | 0.013 | 0.655 | 0.713 |
| rs4148642 | 0.395 | 0.328 | 0.358 | 0.573 | 0.331 | 0.379 | 0.467 | 0.405 | 0.387 | 0.367 | 0.374 | 0.369 | 0.104 | 0.353 | 0.304 | 0.369 | 0.630 | 0.669 | 0.274 | 0.141 | 0.499 |
| rs3758953 | 0.315 | 0.166 | 0.371 | 0.669 | 0.328 | 0.220 | 0.509 | 0.119 | 0.382 | 0.183 | 0.443 | 0.266 | 0.095 | 0.486 | 0.158 | 0.105 | 0.132 | 0.130 | 0.591 | 0.974 | 0.900 |
| rs2188966 | 0.261 | 0.237 | 0.353 | 0.644 | 0.301 | 0.265 | 0.476 | 0.195 | 0.371 | 0.277 | 0.426 | 0.281 | 0.164 | 0.392 | 0.697 | 0.238 | - | 0.234 | 0.415 | 0.927 | 0.670 |
| rs3758947 | 0.934 | 0.836 | 0.918 | 0.858 | 0.928 | 0.994 | 0.982 | 0.915 | 0.978 | 0.943 | 0.978 | 0.943 | 0.927 | 0.992 | 0.433 | 0.486 | 0.967 | 0.102 | 0.237 | 0.437 | 0.577 |
| rs3813002 | 0.227 | 0.222 | 0.314 | 0.744 | 0.349 | 0.183 | 0.569 | 0.012 | 0.295 | 0.144 | 0.336 | 0.176 | 0.175 | 0.422 | 0.125 | 0.196 | 0.953 | 0.013 | 0.222 | 0.682 | 0.222 |
| rs9934336 | 0.724 | 0.724 | 0.729 | 0.679 | 0.714 | 0.635 | 0.698 | 0.635 | 0.695 | 0.657 | 0.691 | 0.701 | 0.665 | 0.725 | 0.586 | 0.719 | 0.917 | 0.794 | 0.714 | 0.998 | 0.753 |
| rs3813008 | 0.491 | 0.593 | 0.505 | 0.637 | 0.545 | 0.526 | 0.521 | 0.605 | 0.488 | 0.520 | 0.496 | 0.465 | 0.507 | 0.588 | 0.607 | 0.551 | 0.891 | 0.343 | 0.776 | 0.537 | 0.320 |
| rs3116150 | 0.149 | 0.172 | 0.226 | 0.677 | 0.293 | 0.186 | 0.479 | 0.138 | 0.279 | 0.162 | 0.341 | 0.203 | 0.165 | 0.342 | 0.150 | 0.241 | 0.883 | 0.626 | 0.442 | 0.728 | 0.167 |
